# Is there a relationship between weather conditions and aortic dissection?

**DOI:** 10.1186/1471-2482-5-21

**Published:** 2005-10-15

**Authors:** Costa Repanos, Neil K Chadha

**Affiliations:** 1 ENT Department, Derriford Hospital, Plymouth, United Kingdom; 2 ENT Department, Torbay Hospital, Torquay, United Kingdom; 3 Blackpool Victoria Hospital, Blackpool, United Kingdom

## Abstract

**Background:**

Bleeding and rupture of blood vessels has been correlated with weather conditions in the past. This is the first study in the world literature with the aim of investigating the relationship between atmospheric pressure and temperature with the presentation of aortic dissection.

**Methods:**

The dates of all emergency aortic dissection repairs from 1996–2002 in a regional cardiothoracic unit at Blackpool Victoria Hospital were obtained. Hourly temperature and pressure data from a regional weather station for this time period was supplied by the Meteorological Office. The mean and standard deviation of hourly temperature and pressure data for that month were compared to the mean and standard deviation of the data 24 and 48 hours prior to the aortic dissection.

**Results:**

26 patients were found to have been operated on during the time period studied. There was no statistically significant correlation between temperature or atmospheric pressure readings, and the incidence of aortic dissection, using a Bonferonni-corrected significance p-value of 0.005

**Conclusion:**

This study is the first to examine the relationship between atmospheric pressure, temperature and dissecting thoracic aorta. No statistically significant relationship was demonstrable.

## Background

Aortic dissection occurs when there is a defect in the intimal layer of the aorta. This causes blood to track through the aortic layers, creating a false lumen between the intima and the overlying adventitia, tending to obliterate the blood supply to branches along its route. The dissection may spread distally to involve the renal, spinal and iliac arteries, or proximally to involve the head and neck vessels, coronary vessels, or aortic root. Once the dissection has occurred it can rupture into the main lumen itself, in which case the patient may survive for some time, or rupture externally leading to haemorrhage and potentially a rapid death.

The known risk factors for aortic dissection are male sex, hypertension, atherosclerosis, diabetes, hyperlipidemia, smoking, syphilis, Marfan's disease, and Ehlers-Danlos disease. In a large series of necropsies of acute aortic dissections more than 40% of patients with proximal dissection died immediately, the rate of death ranged between 1 and 3% per hour. Within 24 hours 70%, within 1 week 94% and within 5 weeks 100% of people with proximal aortic dissection died [1]. After surgical treatment of proximal aortic dissection the survival rate is approximately 70% after 3 years [2].

The aetiology of an acute aortic dissection remains poorly understood. It is thought that the moment of dissection may be precipitated by higher than normal blood pressures, and it has been postulated that variablilty of atmospheric pressure may add to this. There is also a seasonal variation in blood pressure [3,4] and it has been shown that there is a seasonal increase in the rate of non-traumatic aortic dissections in the winter [5,6].

Previous work has observed an increase in ruptured abdominal aortic aneurysms (AAA) in winter [7], and a correlation with atmospheric pressure [8]. Other vascular disruptions, including subarachnoid aneurysm rupture and spontaneous cervical artery dissection, have also been demonstrated to correlate with atmospheric pressure changes [9] or seasonal variations [10].

There have been no previous studies specifically investigating the relationship between weather conditions and the incidence of thoracic aortic dissection. Considering the similarity in seasonal incidence between AAA rupture and aortic dissection, we aim to explore whether there is an association between atmospheric pressure, temperature, and the presentation of aortic dissection.

## Methods

Hospital records were used to identify all emergency aortic dissection repairs between 1996 and 2002 in a regional cardiothoracic unit at Blackpool Victoria Hospital. Data was available from the cardiothoracic minimum dataset, and cases were confirmed by reference to the hospital notes. Ethical approval was obtained to use this data. Temperature and pressure recordings from a nearby regional weather station were obtained for this time period from the United Kingdom Meteorological Office. Blackpool cardiothoracic unit receives referrals from northern Lancashire, and Crosby is the weather station closest to the centre of the unit's catchment area.

The meteorological data used was hourly temperature measurements and hourly atmospheric pressure measurement for a full month centred on the occurrence of each aortic dissection.

Using the meteorological data, the following temperature indicators were calculated (Table 1):

**Table 1:** Temperature indicators

**1**	Mean temperature for the whole month of the operation
**2**	Mean temperature 48 hours before operation date
**3**	Mean temperature 24 hours before operation date
**4**	Mean daily temperature range for the whole month of the operation
**5**	Mean daily temperature range 48 hours before operation date
**6**	Mean daily temperature range 24 hours before operation date

The following pressure indicators were also calculated (Table 2):

**Table 2:** Pressure indicators

**7**	Mean pressure for the whole month of the operation
**8**	Mean pressure 48 hours before operation date
**9**	Mean pressure 24 hours before operation date
**10**	Mean daily pressure range for the whole month of the operation
**11**	Mean daily pressure range 48 hours before operation date
**12**	Mean daily pressure range 24 hours before operation date
**13**	Standard deviation of pressure for the whole month
**14**	Standard deviation of pressure 48 hours before operation date
**15**	Standard deviation of pressure 24 hours before operation date

Indicators were then paired for comparison using each set of monthly data against the corresponding data from 48 hours, and then 24 hours before the operation. The null hypothesis was that there were no differences within each pair of indicators. These hypotheses were tested using paired t-tests and Wilcoxen signed rank tests (depending on the distribution of the data).

The Bonferroni correction is a statistical adjustment to allow for multiple comparisons. A Bonferroni correction was applied by dividing the p-values by the number of outcomes being tested. In this study we have made 10 comparisons between indicators, and have therefore used α = 0.005 for hypothesis testing. The null hypothesis was therefore rejected for p values ≤ 0.005, and the probability of making a Type I error therefore 0.05.

## Results

Data for 26 consecutive operated dissecting aortic aneurysms were obtained for the study time period. Results for the temperature indicators and atmospheric pressure indicators including comparisons between pairs are shown in Table 3 and Table 4 respectively.

**Table 3:** Temperature indicator values and comparison of results.

Indicator A	Mean of A (°C) n = 26	Indicator B	Mean of B (°C) n = 26	Indicator A vs. Indicator B	p-value
					
				Paired t-test (t-value)	
Mean temperature for whole month	11.36	Mean temperature 48 hours before	10.60	-1.7243	0.097
Mean temperature for whole month	11.50	Mean temperature 24 hours before	10.91	1.4658	0.156
Temperature range for whole month	5.94	Temperature range 48 hours before	5.54	0.9972	0.329
Temperature range for whole month	5.82	Temperature range 24 hours before	5.62	0.4275	0.673

**Table 4:** Pressure indicator values and comparison of results.

Indicator A	Mean of A (millibars) n = 26	Indicator B	Mean of B (millibars) n = 26	Indicator A vs. Indicator B		p-value
					
				Paired t-test (t-value)	Wilcoxon signed rank test (z-value)	
Mean pressure for whole month	1011.32	Mean pressure 48 hours before	1009.39	N/A	0.724	0.469
Mean pressure for whole month	1011.32	Mean pressure 24 hours before	1009.88	N/A	0.444	0.657
Pressure range for the whole month	6.99	Pressure range 48 hours before	8.51	N/A	-1.943	0.052
Pressure range for whole month	6.99	Pressure range 24 hours before	8.40	-1.9275	N/A	0.065
Standard deviation of pressure for whole month	2.22	Standard deviation of pressure 48 hours before	2.73	N/A	-2.283	0.022
Standard deviation of pressure for whole month	2.22	Standard deviation of pressure 24 hours before	2.64	N/A	-1.905	0.057

Table 3 and 4 compare both mean temperature and pressure over the month of the event to the mean temperature and pressures respectively over the preceding 24 and 48 hours.

**Figure 1:**
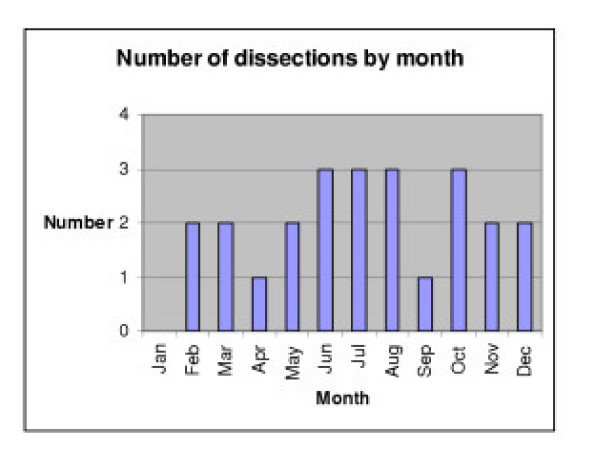
Seasonal variation of aortic dissections (1996–2002).

The distribution of dissections through the year is represented in Figure 1.

Figure 1 depicts the spread of dissections by the month in which they occurred. The numbers are too small to make any comment about seasonality.

## Discussion

Aortic dissection is a condition that presents infrequently to hospitals and is sometimes initially misdiagnosed. Increasingly as hospitals and sub-speciality departments merge to form larger institutions, they will cover a greater population, and the incidence in hospitals will apparently rise.

In our study the most significant result (p = 0.022) showed a trend towards the standard deviation of pressure in the 48 hour period prior to the dissection, being greater than the standard deviation of pressure for that whole month (2.73 vs. 2.22 millibars). This did not reach statistical significance however, after the Bonferroni correction has been applied.

The pressure range was calculated by subtracting the lowest from the highest reading of the day. The mean pressure range for the whole month (6.99 millibars) had a trend towards being lower than the mean 48 hours before dissection (8.51 millibars, p = 0.047) and 24 hours before dissection (8.40 millibars, p = 0.066). This was not statistically significant.

The incidence of aortic dissection had no seasonal or monthly correlation. This is demonstrated by the bar chart (Figure 1). Other studies have demonstrated a seasonal increase in the rate of non-traumatic aortic dissections in the winter [5,6]. The same seasonal variation has been noted in several studies for ruptured abdominal aortic aneurysms (AAA) with an increase in incidence in the winter [7,11,12]. One study found a statistically significant correlation with rupture of AAA and mean atmospheric pressure [8] and the only other published studies found no correlation between the incidence of ruptured AAA and barometric pressure or humidity [7,13]. There have also been studies examining the incidence of other vascular disruptions with changing season and weather conditions. In one study a modest correlation was found between high atmospheric pressure, daily change in pressure and the risk of rupture of subarachnoid aneurysms [9]. Another study demonstrated seasonal variation in spontaneous cervical artery dissection [10].

Previous work has shown that the only physiological parameter positively associated with atmospheric pressure is arterial blood gas concentrations [14]. However the exact mechanism by which reduced arterial oxygen tension may cause rupture of an aneurysm of dissection remains unknown. The onset of labour is the only other medical change thought to be associated with falling atmospheric pressure [15]. Though this possible association may not seem immediately useful, it is known that pregnant women have a higher incidence of spontaneous aortic dissection [16].

The analysis in our study involved using accurate meteorological data and operation dates from a regional cardiothoracic centre covering a large geographical area. Despite careful methodology there are potential errors that must be remembered. The weather station, although centrally placed over the catchment area, may not have reflected the patients' movements over the region in that time period and does not account for weather variations over an area 50 miles in diameter.

There is also likely to be variability in the timing of presentation. Some patients may have begun aortic dissection a long time prior to presenting to hospital and a tamponading effect may have slowed the progression of collapse. We have attempted to account for this by looking at weather data in both the 24 and 48 hour time periods prior to the day of operation. It may be that a sudden change in the indicator being examined between 48 and 24 hours prior to dissection may be related to the onset of dissection.

Only patients who had surgery were included in this study and there are undoubtedly others who either did not survive long enough to have surgery or who were managed without surgery. It is presumed that the inclusion of these patients would not have changed the trends obtained in relation to weather conditions. In practice this study may not have any immediate applications to alter clinical practice, but serves to further elucidate the complex aetiology of a rare and sometimes lethal condition.

## Conclusion

This study is the first to examine the relationship between atmospheric pressure, temperature and dissecting thoracic aorta, and was unable to demonstrate any statistically significant relationship.

## Competing interests

The author(s) declare that they have no competing interests.

## Authors' contributions

CR and NC drafted the manuscript. CR conceived the study. Statistical analysis was performed by ED of the statistics department (see acknowledgements). CR and NC participated in its design and coordination. All authors read and approved the final manuscript.

## Pre-publication history

The pre-publication history for this paper can be accessed here:


